# Reproductive success of Bornean orangutan males: scattered in time but clustered in space

**DOI:** 10.1007/s00265-023-03407-6

**Published:** 2023-12-06

**Authors:** Maria A. van Noordwijk, Laura R. LaBarge, Julia A. Kunz, Anna M. Marzec, Brigitte Spillmann, Corinne Ackermann, Puji Rianti, Erin R. Vogel, S. Suci Utami Atmoko, Michael Kruetzen, Carel P. van Schaik

**Affiliations:** 1 Department of Evolutionary Anthropology, University of Zurich, Zürich, Switzerland; 2 Comparative Socio-Ecology Group, May Planck Institute of Animal Behavior, Konstanz, Germany; 3 Institute des Sciences de l’Evolution Montpellier, University of Montpellier, Montpellier, France; 4 Division of Animal Biosystematics and Ecology, Department of Biology, IPB University, Bogor, Indonesia; 5 Primate Research Center, IPB University, Bogor, Indonesia; 6 Department of Anthropology, Center for Human Evolution Studies, Rutgers, The State University of New Jersey, New Brunswick, USA; 7 Fakultas Biologi, Universitas Nasional, Jakarta, Indonesia; 8 Center for the Interdisciplinary Study of Language Evolution (ISLE), University of Zurich, Zürich, Switzerland

**Keywords:** *Pongo pygmaeus*, Paternity, Male reproductive careers, Male bimaturism, Range use, Infanticide

## Abstract

**Abstract:**

The social and mating systems of orangutans, one of our closest relatives, remain poorly understood. Orangutans (*Pongo* spp*.*) are highly sexually dimorphic and females are philopatric and maintain individual, but overlapping home ranges, whereas males disperse, are non-territorial and wide-ranging, and show bimaturism, with many years between reaching sexual maturity and attaining full secondary sexual characteristics (including cheek pads (flanges) and emitting long calls). We report on 21 assigned paternities, among 35 flanged and 15 unflanged, genotyped male Bornean orangutans (*Pongo pygmaeus wurmbii*), studied from 2003 to 2018 in Tuanan (Central Kalimantan, Indonesia). All 10 infants born since mid-2003 with an already identified sire were sired by flanged males. All adult males ranged well beyond the study area (c. 1000 ha), and their dominance relations fluctuated even within short periods. However, 5 of the 10 identified sires had multiple offspring within the monitored area. Several sired over a period of c. 10 years, which overlapped with siring periods of other males. The long-calling behavior of sires indicated they were not consistently dominant over other males in the area around the time of known conceptions. Instead, when they were seen in the area, the known sires spent most of their time within the home ranges of the females whose offspring they sired. Overall, successful sires were older and more often resident than others.

**Significance statement:**

It is difficult to assess reproductive success for individuals of long-lived species, especially for dispersing males, who cannot be monitored throughout their lives. Due to extremely long interbirth intervals, orangutans have highly male-skewed operational sex ratios and thus intensive male-male competition for every conception. Paternity analyses matched 21 immature Bornean orangutans with their most likely sire (only 10 of 50 genotyped males) in a natural population. Half of these identified sires had multiple offspring in the study area spread over periods of at least 10 years, despite frequently ranging outside this area. Dominance was a poor predictor of success, but, consistent with female mating tactics to reduce the risk of infanticide, known “sires” tended to have relatively high local presence, which seems to contribute to the males’ siring success. The results highlight the importance of large protected areas to enable a natural pattern of dispersal and ranging.

**Supplementary Information:**

The online version contains supplementary material available at 10.1007/s00265-023-03407-6.

## Introduction

 Socioecology generally predicts a correlation between a species’ social system and its mating system, or more precisely the individual reproductive careers of both females and males (van Noordwijk and van Schaik 2004; Schülke and Ostner 2012). To measure variation in lifetime reproductive success and detect the sources of such variation, we ideally monitor individuals throughout their lives (e.g., Foroughirad et al. 2022). However, such monitoring must overcome various hurdles, especially in species with a long lifespan. In most animals, individuals of at least one sex disperse from the natal area and their relatives (Greenwood 1980; Trochet et al. 2016), making it hard to keep track of dispersing individuals and assess their reproductive success, especially when they move over large distances (Bartoń et al. 2019). In most mammals, males are the dispersing sex, making this problem most acute for them. For males, we also face a second hurdle, because assessing their reproductive success requires genetic analyses, whereas identifying all potential sires of a female’s offspring is often difficult. Males may transfer into unmonitored social units (e.g., hyenas (*Crocuta crocuta*): Curren et al. 2022; macaques (*Macaca fascicularis*)*:* van Noordwijk and van Schaik 2001; capuchins (*Cebus capucinus*): Jack and Fedigan 2004). Likewise, non-monitored “extra-group” males may sire offspring (e.g., Isvaran and Clutton Brock 2007*;* Ostner et al. 2008; Ruiz-Lambides et al. 2017), or males may defy detection by individually roaming over huge areas (e.g., sperm whales (*Physeter macrocephalus*): Whitehead and Weilgart 2000; elephants (*Loxodonta africana*): Hollister-Smith et al. 2007; giraffes (*Giraffa camelopardalis*): Castles et al. 2019; Muller and Harris 2022; black bears (*Ursus americanus*): Costello et al. 2009; polar bears (*Ursus maritimus*): Derocher et al. 2010; Richardson et al. 2020).

These challenges explain why our understanding of the social and mating systems of orangutans (*Pongo* spp.) remains incomplete. Females are philopatric and maintain differentiated social relationships in “neighborhoods” (Singleton and van Schaik 2002; Wich et al. 2004; Knott et al. 2008; Morrogh-Bernard et al. 2011; van Noordwijk et al. 2012, 2018; Ashbury et al. 2020), albeit without forming delineated social units (van Schaik 1999). Males, however, disperse on both Sumatra (*Pongo abelii*) and Borneo (*P. pygmaeus*) when 12–15 years old and often move far from their natal area (Morrogh-Bernard et al. 2011; Nater et al. 2011; Arora et al. 2012; Nietlisbach 2012). Once dispersed, they range individually over extensive areas, with documented estimates of up to 4000 ha, which is much larger than any current study site (Galdikas 1988; Setia et al. 2009; Singleton et al. 2009; Utami Atmoko et al. 2009b; Buckley 2014). Moreover, variation in male presence in study areas and in the duration of their “absences” has led many to assume a differentiation in resident and roaming males (Utami Atmoko et al. 2009a; Buckley 2014; Spillmann et al. 2017b). Complicating matters, males also show bimaturism: once sexually mature, the development of their distinctive secondary sexual characteristics (cheek pads known as flanges and large throat pouches that enable the production of loud vocalizations known as long calls) tends to be delayed for a period of 10 years or (much) longer after dispersal (Utami Atmoko et al. 2009a), leading to two adult morphs known as unflanged and flanged males with different mating tactics (Utami Atmoko and van Hooff 2004; Dunkel et al. 2013; Kunz et al. 2023).

Consequently, for natural populations, we lack documentation of which males reproduce where and when, despite some reports on siring success in areas with fragmented forest or including reintroduced individuals (e.g., Utami Atmoko et al. 2002; Goossens et al. 2005, 2006; Banes et al. 2015; Tajima et al. 2018). This lacuna is especially unfortunate from a comparative perspective. Orangutans are great apes and therefore among our closest living relatives. The factors affecting male reproductive success and thus shaping the social systems of most of the African great apes are better understood (Watts 2012). They show considerable variation in patterns of dispersal, social unit size, and stability, as well as the role of dominance in reproductive success and age of peak reproductive rates (e.g., chimpanzees (*Pan troglodytes* sp*.*): Langergraber et al. 2012, 2013; Muller et al. 2020, bonobos (*Pan paniscus*): Surbeck et al. 2017; Ishizuka et al. 2018, gorillas (*Gorilla* sp*.*): Robbins and Robbins 2018; Manguette et al. 2020; Masi et al. 2021). Reliable data on patterns of orangutan reproductive success would improve our picture of great ape social and mating systems and thus our understanding of patterns in hominid and hominin social evolution.

We assessed paternities of Bornean orangutans (*P. p. wurmbii*) in a c. 1000-ha study area within a much larger peat-swamp habitat (Tuanan, Central Kalimantan). Although our study covered 15 years and included over 150 recognized individuals (Table 1), it remains preliminary, because orangutans may live for over 50 years, and we expect age-dependent variation in male reproductive tactics and success (Knott et al. 2010; Kunz et al. 2021b, 2023). We can, however, estimate the distribution of assigned paternities among males in the same and different stages of their lives and thus explore reproductive career trajectories.

**Table 1:** Overview of all recognized individuals per age-sex class seen in the study area and whether genetic material was available: Only those with 15–20 scored loci were included in parentage analyses (in bold), whereas unique identity was established for individuals with 9–14 scored loci. Individuals with no established genotype were recognized based on visible features, whereas immatures were identified through consistent association with their mother. The numbers are given for the individuals’ age class by the end of the study in 2018

Last age class	No genotype	Unique, insufficient genotype	Unique, sufficient genotype	Total # known
Parous female	1	1	**26**	28
Nulliparous fem.			**6**	6
Independent imm.	2	1	**18**	21
Dependent imm.	14	2	**9**	25
Flanged male	6	10	**35**	51
Unflanged male	9	6	**15**	30
Total	32	20	**109**	161

Although work on paternities in natural populations of orangutans is scarce, behavioral observations suggest we should expect only moderate reproductive skew. First, males do not defend territories, and females mate with numerous unflanged and flanged males prior to each conception (Fox 2002; Knott et al. 2010; Kunz et al. 2022). Males may have difficulty identifying reproductive opportunities due to the combination of the very long interbirth intervals of orangutans (average 7.6 years: van Noordwijk et al. 2018), asynchronous births, and especially the absence of females’ signaling ovarian activity (Nadler 1995; Knott et al. 2010; Durgavich et al. 2022). Moreover, because male-female association is energetically rather costly (Kunz et al. 2021a), long-term mate guarding is impossible, making it unlikely that any male can monopolize conceptions.

Males nonetheless clearly compete for mating access. Through their long calls, they inform conspecifics about their location, potentially attracting females while repelling rivals (Mitani 1985; Setia and van Schaik 2007; Askew and Morrogh-Bernard 2016; Spillman et al. 2017b). In general, locally dominant and confident males call more frequently and also respond more often to calls by other males (Setia and van Schaik 2007; Buckley 2014; Spillmann et al. 2017b). This should favor dominants in mating competition. Reports on NW Sumatran orangutans tend to report long-term stability in male dominance (Schuermann and van Hooff 1986; Fox 2002; Utami Atmoko et al. 2002). However, no such pattern is apparent on Borneo (Dunkel et al. 2013; Buckley 2014; Spillmann et al. 2017b), where long intervals between encounters by rivals (Spillmann et al. 2017b) and greater fluctuation in body condition (Knott 1998; O’Connell et al. 2021) seem to preclude a stable hierarchy. Thus, although a male’s long-calling behavior could at least indicate his temporary confidence level and willingness to engage in a confrontation, dominance relationships seem to fluctuate fast enough that a strong reproductive skew is unlikely (Spillmann et al. 2017b).

Thus far, most comparisons of siring success concerned the difference between flanged and unflanged males. The latter are at a disadvantage whenever a flanged male is in proximity to a female (Kunz et al. 2021b) and throughout the tenure of a resident dominant flanged male (Utami Atmoko et al. 2009a; Banes et al. 2015). Moreover, females are reported to prefer mating with fully flanged males (e.g., Schuermann and van Hooff 1986; Banes et al. 2015), especially around the time when conception is likely (Knott et al. 2010). Accordingly, flanged males are expected to sire most of the offspring, whereas unflanged males are thought to only have some siring chances with nulliparous females, whose fertility is very unpredictable, as well as during periods of dominance instability among flanged males (Utami Atmoko et al. 2009a). Nonetheless, data from non-provisioned natural populations are scarce and the hypothesized reproductive advantage of the switch from “unflanged” to “flanged” morph, the extent of its delay, and how it relates to monopolization potential (Pradhan et al. 2012) still need further documentation.

Here, we examine which males in this non-territorial, non-gregarious, long-lived species achieved reproductive success and when. We identified sires based on genetic parentage analyses and constructed a profile of successful males based on their morph, the frequency of their presence in the area, and long-call behavior as an indication of their confidence, as well as their space use while they are in the study area.

## Methods

### Field methods

This study on orangutans (*Pongo pygmaeus wurmbii*) was conducted at the Tuanan Research area (2° 09′ South; 114° 26′ East) between July 2003 (after about 1 year of informal observations) and July 2018. The total study area (hereafter referred to as “Tuanan”) has a grid of trails encompassing c. 10 km^2^ and is located at the southwestern edge of the c. 3000-km^2^ Mawas Conservation Area (Central Kalimantan, Indonesia). Despite increasing degradation at the edges, orangutans moved freely into and out of the large adjacent forest area. The study site consists of recovering formerly selectively logged peat-swamp forest and has an orangutan density of ± 4.3 individuals/km^2^ (van Schaik et al. 2005). Once located, habituated individuals were followed by 1–3 well-trained observers for multiple (max. 10) consecutive full “nest-to-nest” days within the study area, allowing at least 4 weeks until the next focal period for that individual (cf. van Noordwijk et al. 2012; Vogel et al. 2017; Ashbury et al. 2020; see full methods: https://www.aim.uzh.ch/en/research/orangutannetwork/sfm.html). During this study, we collected 18’022 h of focal observation data on flanged and 6’977 h on unflanged males; and around 75’000 h on other age-sex classes, during which we also made *ad lib* observations on male presence and male-male interactions.

Individuals were considered to be in “association” when they were within 50 m of each other, and “encounter” marks the onset of an association irrespective of its duration.

### Individual recognition

Consistent individual recognition was challenging because of the long intervals (sometimes even multiple years) between encounters with individuals, especially males. Since any form of marking or tagging of orangutans is unethical and prohibited, we relied on two methods. First, we achieved visual identification through photos of facial and bodily features, paying special attention to facial scars and damaged fingers and toes (cf. Dunkel et al. 2013; Spillmann et al. 2017b). Since 2003, photos of focal individuals, and, when possible, conspecifics in association, were taken opportunistically and since 2012 systematically during every focal follow day. Male identities were consistently verified by at least two among AM, BS, JK, and MvN using all available photos. Second, we combined and updated photo-identification with genetic results (see below) whenever available. Still, we could not prevent some uncertain identifications in the field, especially of rarely encountered males. Dubious identifications were excluded from all analyses. Most immatures could be assigned unambiguously to an adult female as mother, either because they were seen being nursed during the study period or because they were of pre-dispersal age and still occasionally associated peacefully with this female; in the latter case, genetic maternity analyses were used to confirm these assignments (see below). Because our study involved focal animals in the field, we could not collect data blindly.

### Presence in the area

To estimate “presence,” we recorded all sightings of identified individuals anywhere in the area on a daily basis (whether it was a focal individual, in association with a focal individual, or independently encountered, following Singleton and van Schaik 2001). Presence data were combined in a 1/0 score per calendar month, as the number of observers and most intensively visited locations varied over time. Because individuals can easily be missed due to their habits and our focus on focal follows, we lumped these monthly records into 3- or 6-month bins to provide a rough minimum estimate of their presence in Tuanan during the study period. Since results for these bin sizes were similar, we focus on the more conservative 6-month bins. We considered a male a “resident,” when he had been seen during at least one 6-year block in at least 67% of the half-year periods, a “visitor” if he had been seen only in one half-year period or in < 10% of the half years after first being recognized, and otherwise as an “irregular.” We chose the 6-year blocks to cover the period of strongest infant dependence. If we relax this to 4 years, we add more paternities to “long-term resident” males, strengthening the outcome presented in the results.

Focal follows could not always be maintained over at least 5 days. Males, in particular, were lost significantly more often because they traveled rapidly on the ground, often after an encounter with another orangutan (Ashbury et al. 2015), or because they left the study area (Table S1). Moreover, when females left, they did so in a consistent direction (Ashbury et al. 2020), whereas multiple males were seen to leave the area both to the north and the south and were mostly not seen for longer time periods (weeks or even years), consistent with ranges far exceeding the size of the study area (cf. Singleton and van Schaik 2001; Buckley 2014).

### Presence during “conception windows”

A male’s association with one of the resident females is a precondition for siring her offspring. On average, unflanged males spend almost twice as much time in association with nulliparous and parous females compared to flanged males in Tuanan (Kunz et al. 2021a), and they also encounter females on more than twice as many days (Table S2). However, due to long gaps between follows of focal individuals (usually > 6 weeks), our data are unlikely to capture female-male association time at the most likely time of conception. Therefore, we use confirmed “presence” in the area as a minimum indication of a male’s chance to have sired an offspring.

The birth month was known or estimated within 3 months (cf. van Noordwijk et al. 2018) for 8 infants with an assigned sire. To assess which males had had the opportunity to sire these infants, we compiled which males were “present” in at least 1 month of the “conception window” (Table S3), which we defined as between 4 months before conception and 2 months into pregnancy (assuming a gestation period of ca. 8 months: Sodaro et al. 2007), i.e., 12–6 months before the known or estimated birth month.

### Male dominance relationships

The number and combinations of males present in the area were highly variable, because all males spent time outside Tuanan, with large variation in the duration, frequency, and intervals between the periods of their presence. Overall, the number of recorded agonistic interactions among males was too low for a meaningful dominance rank analysis over the entire study period: In 15 years, a total of 132 agonistic interactions within visible range were witnessed in which both male participants (of either morph) were identified, involving only 41 (of 81) different males. Moreover, except for flanged males being consistently able to displace unflanged males, dyadic relationships among flanged males were found to be unstable (Spillmann et al. 2017b).

Long calls are only emitted by flanged males and have distinct acoustic features (Spillmann et al. 2010, 2017a; Askew and Morrogh–Bernard et al. 2011). In the flat peat-swamp forest of Tuanan, long calls can be heard over at least 1300 m (Spillmann et al. 2017b) and preserve the caller’s characteristics over at least 300 m (Lameira and Wich 2008), enabling males to avoid unwanted close encounters. However, since human observers could rarely identify the identity of a distantly calling male, these cannot be used for dyadic avoidance or inferred dominance analyses. Yet, whenever a focal male responds to another male’s long call by approaching, counter-calling or avoiding, potentially leading to a confrontational assessment (Spillmann et al. 2017b), we can assess how confident the focal male is. In contrast, if he does not respond, this may also reflect his unwillingness to attract eavesdropping males who he may have encountered or heard recently. We used the rates of emitting long calls and responding with a long call to a long call heard (if estimated to be within 500 m) as a measure of a male’s confidence or “self-assessed relative dominance” at that moment (cf. Spillmann et al. 2017b).

### Ranging

During all focal follows, coordinates were recorded every half hour: initially on hand-drawn maps, and since 2012 by collecting waypoints on hand-held GPS units. Garmin MapSource software (v6) and ESRI’s ArgGIS (v9.3) software were used to digitize hand-drawn maps and to import GPS unit data. These data were all collected inside the study area, and thus do not reflect the complete ranges of adult males, who all spent time outside Tuanan as well (see presence data). Here, we focus on how the ranges of assigned sires relate to the ranges of their known local female partners to assess how this affects their siring success (Table S4).

To examine whether known sires concentrated their long-term space use near mates, we fit and selected continuous-time stochastic models (via AICc) to individual movement processes of adult female and flanged male orangutans (based on individual ranging data). We then used these to create autocorrelated kernel density estimated utilization distributions (AKDEs) representing their space use within Tuanan. These were weighted to deal with irregular sampling schedules (Fleming et al. 2018). We limited analysis to those sires (*N* = 4) that displayed evidence of long-term range residency during the 2003–2018 period, as indicated by visual inspection of semi-variograms (Calabrese et al. 2016; Noonan et al. 2019). We then used these AKDEs to quantify each sire’s distributional overlap with mate and non-mate adult females via a Bhattacharyya coefficient (BC) corrected for small sample sizes. BC values compare two continuous utilization distributions (and thus differ from most other home range overlap values) and range from 0 to 1, with 0 representing completely independent distributions, and 1 representing identical distributions (Winner et al. 2018). Associated confidence intervals around the BC point estimate are entirely positive and capture the uncertainty in the AKDE calculation (Winner et al. 2018). In the four instances where mother-sire pairs were sampled enough to meet range residency, we also compared BC values between mates in the 3 years pre- and post-birth to see if males changed their overlap when mates had dependent infants. We used the R package “ctmm” for this analysis (Calabrese et al. 2016; R Core Team 2021; see script in ESM 4). In addition, we calculated the absolute spatial area of overlap of the 95% contours of their home ranges following Tilberg and Dixon (2022).

### Genetic analyses

**Fig. 1:**
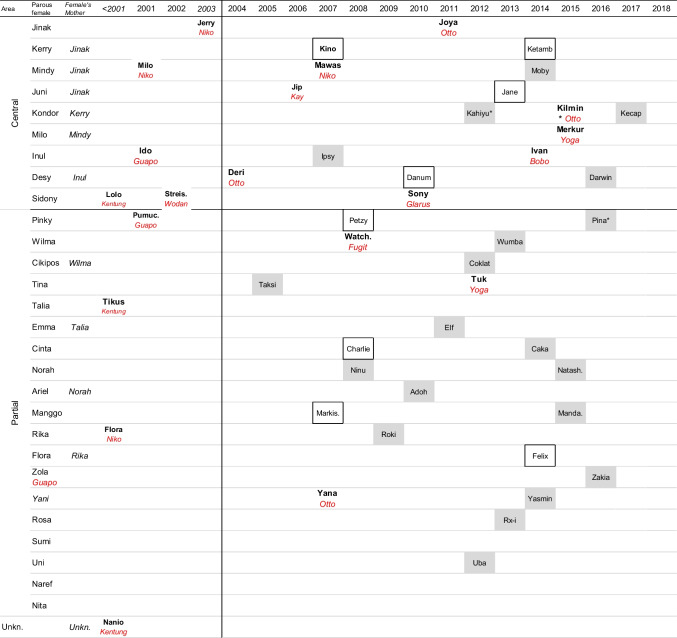
Overview of all known offspring per female per birth year during the study (the study started mid-2003; ages of immatures present were estimated per year or “born <2001”). Assigned sires are indicated under infant’s name in red, for as far as available. Offspring without assigned sires despite sufficient genotype for the immature are indicated in boxes, and offspring without sufficient genotype are indicated in gray shading. Mothers ranging completely within the core study area are labeled as “central,” those ranging also outside this area as “partial,” for one adolescent male a sire but no mother was found. *These infants did not survive to 2 years

Fecal samples were collected non-invasively and opportunistically. We followed the same laboratory protocol for DNA extraction from fecal material as used for the initial genetic analyses by Arora et al. (2010, 2012), including using the same autosomal microsatellite markers (Table S5), using Cervus 3.0.7 (www.fieldgenetics.com; based on Kalinowski et al. 2007) for further analyses. We expanded the initial dataset by Arora et al. (2012) from 49 to 109 individuals with a genotype based on 15–20 markers (see Table 1: 26 of 28 parous, all 6 nulliparous females, 50 of 81 known post-dispersal males, and 27 of 46 known immatures). Unfortunately, 52 of 161 recognized individuals could not be sufficiently genotyped with at least 15 scored loci, including 16 offspring of known mothers (Table 1, Fig. 1), mostly due to a lack of samples. The majority of the 31 males with an insufficient genotype were rarely seen in the area. Visual inspection revealed that all genotypes were unique, as all individuals differed at 7 loci at least. In addition, haplotypes were assessed to supplement both maternity and paternity results whenever available: Mitochondrial (mtDNA) haplotypes were based on distinct sequences in 450 bp of the hypervariable region I of the mtDNA (following Arora et al. 2012). Y-chromosomal haplotypes were based on 18 markers (Nietlisbach et al. 2012 and additions by Fluck 2019), assessed only for 27 adult and 4 immature males (see ESM 2, 3).

We used Cervus (version 3.0.7) for the parentage analyses, including only individuals with at least 15 shared genotyped loci for the particular parent-offspring dyad, as well as for the mother-offspring-sire trios. In the maternity analyses, only potential mothers with the same mtDNA haplotype as the focal individual were included. For paternity analyses, known mothers were included whenever possible. For all parentage analyses we allowed a 5% error rate, and 10% of potential parents sampled, and parentage was assigned with a strict 95% confidence level. All potentially post-dispersal males (ranging independently without a known or assigned local mother) were included as potential sires, irrespective of their flanging status at the time of an offspring’s estimated conception or whether they had been sighted around this time.

We found that the confidence of the maternity assignments for known mother-offspring dyads was reduced by the presence of first-degree maternal relatives (ESM 2). Therefore, we checked for the presence of father-son dyads among the adult males seen in the area (ESM 2) to assess the likelihood that the standard procedure would yield similar problems with paternity assignments. As did Arora et al. (2012), we found that adult males were much less likely to have a close relative in the same area. We therefore ignored the presence of possible close male relatives as a source of error in our paternity analyses of immatures with known mothers.

## Results

### Paternity assignments

**Fig. 2:**
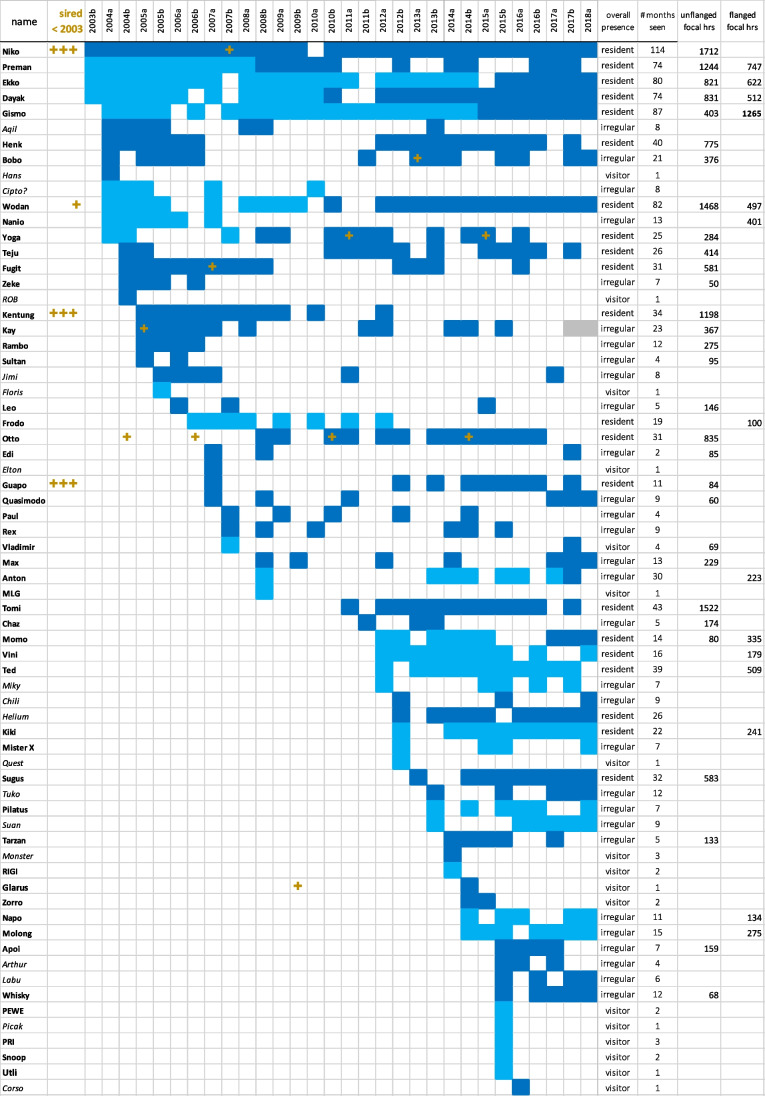
Presence per half year of all recognized males and when they are known to have sired an offspring indicated by +. Overall presence class: “residents” are seen at least in one 6-year block in at least 67% of the half-year periods, “visitors” have been seen only in one half-year period or in < 10% of the half years after first being recognized, and otherwise, males are classified as “irregular.” Flanged morph in dark blue; unflanged morph in light blue; name in bold: genotype included in paternity analyses

A high-confidence sire-offspring-mother trio was assigned for 18 offspring (Table S6). For an additional 3 individuals (first known as pre-reproductive), only a sire was assigned, because the mother was not sampled or identified (Table 2). Fig. 1 shows all 21 individuals with assigned sires. Only 11 of these were conceived during the study, the other 10 before observations started mid-2003. Two maternal sisters, with an estimated interbirth interval of c. 7 years, were assigned to the same sire. Possibly, there is a second pair of full siblings, but for the younger one, we had insufficient genetic data (13 microsats) for inclusion in the high-confidence assignments. Based on estimated ages of offspring sired before 2003, the assigned paternities of some males are spread out over 10 years or more, overlapping with such siring periods of several other males in Tuanan (Fig. 2).

**Table 2:** Number of offspring sired per male morph: offspring sired before the start of the study (July 2003) and those sired during the study with assigned sires (see also Fig. 1). In brackets, the number of offspring by known primiparous mothers

	Morph of sire	Mother known^#^	Mother unknown*	Total
Sired before 2003	Flanged			10
	Unflanged	1		
	Unknown morph	7	2	
Sired after mid-2003	Flanged	8(3)	1	11
	Unflanged			
	Unknown morph	2 (1)		
Total		**18**	**3**	21

#Trio consisting of (confirmed) genotyped mother–offspring–assigned sire

*Paternity only for young individuals without genotyped mother

Unfortunately, we could not assign a sire to 8 of 19 genotyped infants born during the study. A sire was assigned with high confidence (Fig. 1) to only 8 of 12 (67%) offspring, with a mother ranging mostly within Tuanan and to 3 of 7 (43%) with a mother ranging only partially in the study area (i.e., for whom we may have missed potential sires).

### Paternity and bimaturism

For 10 of the 19 genotyped infants born during the study, we could assign a sire with known flanging status. In all 10 cases, the sire was known to be flanged at the time of conception (Figs. 1 and 2). One infant born before the start of the study was sired by a male who was still unflanged during the early years of the study, showing that unflanged males can sire offspring. For the other infants with assigned sires, the flanging status of the sire was unknown. However, several of the successful sires before 2003 were estimated to be older flanged males at the start of the study.

We could not test whether unflanged males were more successful with nulliparous females, as suggested in the literature. However, the one paternity by an unflanged male was with a parous female, whereas the assigned sires of the first infant of three primiparous females were flanged.

**Fig. 3:**
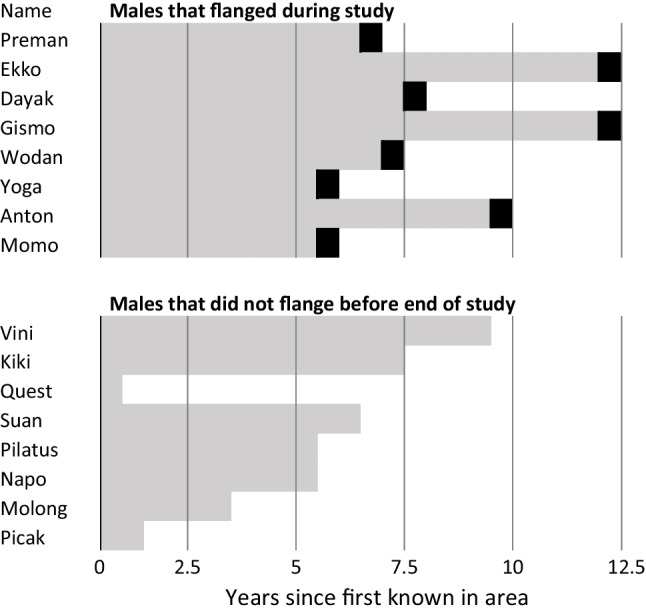
Number of years since individual males were first recognized in the study area before they grew flanges. The top panel shows males that grew flanges during the study (black boxes indicate the flanging period). The lower panel shows males that had not yet grown flanges when the study ended. The figure only includes males with genetic identification and that were at least as large as an adult female when first encountered and thus estimated to be at least 15 years old at “year 0” in the graph (males are listed in the same order as in Fig. 1)

During this study, 8 males, initially known as unflanged, changed into the flanged morph (confirmed identification for both morphs). For males that were at least as large as adult females (estimated to be over 15 years old) when first named, Fig. 3 shows the (left-censored) intervals until they flanged. It also shows the left- and right-censored durations for those still unflanged at last sighting. This small sample suggests that males tend to flange c. 10 years after reaching female size and thus when they are approximately 25 years old in this population.

All 8 males known to have flanged during the study showed signs of physical fights (fresh facial scars, mutilated fingers/toes) within a year of flanging. Yet, despite their active involvement in male-male contests, only one was assigned as sire of two infants: he sired one infant born 4 years and another 7 years after he had flanged. Three other males, present much more often, did not sire any offspring in the area within the first 7, 5, and 5 years after flanging, and two apparently did not sire during the first 2 years after flanging (all numbers until the end of the study period). The last two to grow into the flanged morph had had no opportunity to sire any known infants yet. Overall, a male’s siring success is very low during the unflanged state and does not rise to a clear peak immediately after flanging.

### Paternities and presence

**Fig. 4:**
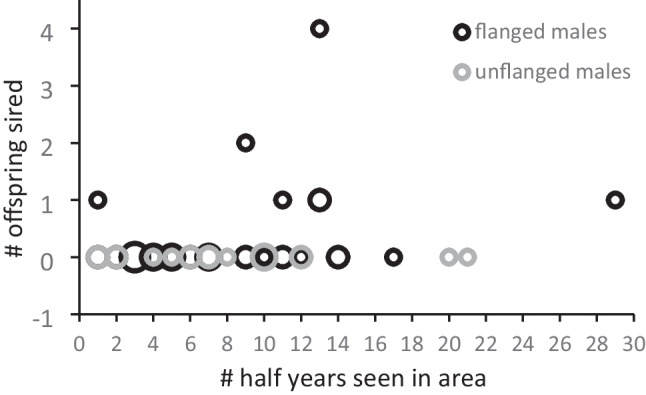
Total observed presence per half year (top: max *N* = 30: Jul 2003–July 2018) of all recognized flanged males in the study area in relation to the number of assigned offspring sired during the study period. The size of the bubble indicates number of identical values. See Fig. S1 for presence per 3-month periods (max *N* = 60)

Fig. 2 shows the total observed presence of all recognized males in Tuanan (Jul 2003–July 2018: max *N* = 30). The half-year presence score correlates with the number of assigned offspring, but not very strongly (Fig. 4: for flanged males: *r*_s_ = 0.35, *t* = 2.15, *P* = 0.039; lumping presence data per 3 months yields a weaker correlation: Fig. S1: *r*_s_ = 0.29, *N* = 35, *t* = 1.77, *P* = 0.086). Similarly, relatively more of the “residents” were identified as sires of a local infant than “irregulars” and “visitors.” Even if the 4 infants of central females without an assigned sire were the offspring of one of the insufficiently genotyped irregular residents or visitors, their per capita success would be lower than that of residents (8 infants for 13 resident flanged males vs. 3 + 4 for at least 22 irregulars + 8 visitors). Although many unflanged males have not had the chance yet to be counted as residents, 4 of 5 males who were residents when unflanged remained resident after flanging (the exception was still often but irregularly seen after being wounded multiple times). Thus, at least some males settled and concentrated their roaming in a relatively limited area (albeit still larger than the known study area) within a few years of natal dispersal over unknown distances.

All but one of the 8 sires were seen during the 6 months surrounding the conception of the infant (Table S3). The exception was not recognized yet at the time and thus might have been labeled in the records as an unidentified male. This confirms that males may occasionally visit Tuanan without being recorded and that our monthly presence data should be regarded as a minimum indication of whether a male was present in the area.

### Paternity and long-call rates

Over the 2003–2018 period, focal flanged males emitted on average 2.7 ± SD 3.4 long calls/day (range 0–20; *N* = 26 identified males with at least 10 fully observed nest-to-nest days, *N* = 1021 full days). No long calls were given by these males on 36.0 ± SD 24.4% of their focal days, and no male called every day. To assess whether observed long-call rates by males present during the local conception windows reflected their likelihood of siring offspring, we considered males for whom we had at least 50 h of focal data during the relevant periods. For 4 of the 8 conception windows, we had such data for flanged sires as well as 1–6 other flanged males, but never all sighted males, permitting long-call rate comparisons between sires and some non-sires. In only 2 of the 4 periods did the assigned sire have a higher long-call rate than the other flanged focal males during the same conception window, whereas in the other two periods, 1 resp. 2 non-sire males called more often. Even though the data are too sparse for further meaningful analyses, this suggests that long-call rates alone do not predict a male’s siring chances within the conception window during which he is present.

The data on response to long calls are even more sparse: In the year a male is known to have sired, he did not counter-call more to the long calls he heard than other flanged focal males (sires responded with a long call to 3 out of 32 heard long calls vs. 113 out of 1370 by non-sires: Kolmogorov Smirnov *D*_max_ = 0.003, *P* > 0.10; based on only long calls estimated to be emitted within c. 500 m) and hence did not appear more confident than all other males present. Despite our very small sample size on long calling by individual males, sires did not distinguish themselves as being clearly more confident by emitting long calls or responding to them more frequently.

### Paternity and ranging

**Fig. 5:**
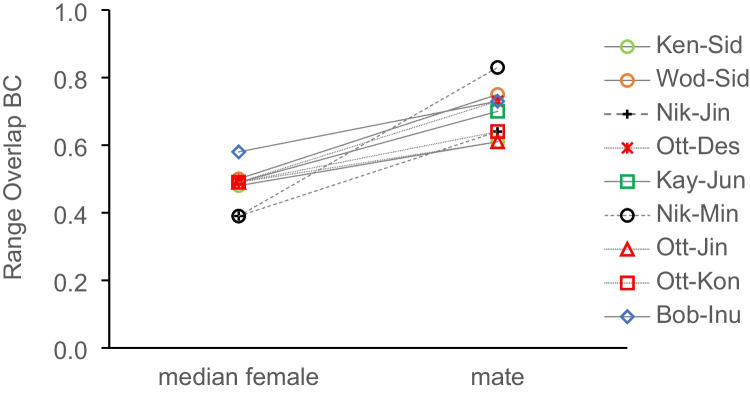
Pairwise distributional range overlap of female-male dyads, based on AKDEs (autocorrelated kernel density estimates) calculated via the Bhattacharyya coefficient (BC), the expected overlap from female and male perspective based on the medians for all range-resident members of the opposite sex, and the percentage of each individual’s range covered by the area of overlap with the mate (see Table S6 for confidence intervals). Note: offspring of dyads Ken-Sid and Wod-Sid were born before 2003 and > 5 years before first range data were collected on female Sid

Most males, including the identified sires, were seen to leave Tuanan regularly. Yet, the sires’ long-term space use within the study area (Fig. S2) was uneven. The sires of 7 infants born during the study were all found to have more range-use overlap with the mothers of these offspring than expected based on the median pairwise overlap with all females (Fig. 5; Table S7; see Fig. S3 for long-term female ranges). This indicates that these males concentrated their siring and space use in a relatively small area (Fig. S4) within their known range inside the study area and mostly encompassed their mates’ (much smaller) ranges. From the female’s perspective, their median expected overlap with all males (sires and non-sires) is relatively high and not different for sires and non-sires (Table S7). This suggests that males may be choosing to spend time around potential mates, while females, with smaller home ranges, are not doing the same.

In the four examples for which we could compare the intensity of space use during a 3-year period before and after the birth of the sired infant, we found that sires maintained spatial overlap with these females in both periods. However, there was no evidence that sires tended to increase overlap with the mother-infant dyad post-birth (e.g., BC values and associated confidence limits were largely similar before and after; Fig. S5; Table S8). Male ranges, including those of all known sires (Fig. S6), overlapped with one another in space, but because this calculation was made over a long period of time and males would leave and return to Tuanan, this result does not preclude spatiotemporal avoidance.

## Discussion

Among diurnal primates, orangutans, especially Bornean ones, are unusual in that females spend most time only with their own (semi-)dependent offspring and form loose neighborhoods, with an individual-based fission-fusion association pattern within stable individual home ranges (van Schaik 1999; Arora et al. 2012; van Noordwijk et al. 2012;﻿﻿﻿﻿ Ashbury﻿﻿﻿ et al. 2020). In contrast, mature males range over much larger areas seemingly without clear boundaries and overlapping with numerous other males (Singleton et al. 2009; Utami Atmoko et al. 2009b; Buckley 2014). Our genetic analyses assigned a sire to 21 immatures, including 11 infants born during the study, since neither all infants nor all potential sires, including those seen in association and mating with the respective mothers, could be genotyped. Strikingly, flanged males sired almost all offspring and several males sired offspring over a period of c. 10 years in a relatively small part of the study area. It is possible that some of the as yet non-assigned males did sire one of the local infants that could not be sufficiently genotyped or offspring born elsewhere. Similarly, we cannot exclude that the local Tuanan sires have additional offspring both inside and outside the limited study area. Therefore, we focus here on what we do know about the known sires.

Overall, the current data suggests at least moderate instantaneous and lifetime reproductive skew among the males, although they live too long for us to estimate lifetime reproductive success of individuals. We also cannot exclude the presence of alternative reproductive tactics among the males even within the two morphs (but do not have enough data to meaningfully discuss these). In this discussion, we use our results to explore the factors affecting male siring success, especially bimaturism, as well as the tactic used by residents.

### Bimaturism

The unusual delay in the acquisition of full secondary sexual characteristics (bimaturism) of orangutan males should only have evolved if both morphs have context-specific advantages. In this study, all genotyped infants born during the study period with an identified sire were sired by flanged males. Nevertheless, one of 10 infants born in the years before 2003 was certainly sired by a male still unflanged at the time (Wodan), whereas all other sires were already flanged when first identified. Based on our observation that only one of four well-known newly flanged males sired a known offspring within the first 5 years after flanging, we consider it unlikely that more than 1–2 of these sires were still unflanged. An unbiased estimate of the percentage of infants sired by flanged males should therefore be at least 90%. This result corroborates findings in studies on populations with less natural conditions (e.g., Utami Atmoko et al. 2002; Goossens et al. 2006; Banes et al. 2015).

The siring bias toward flanged males is not surprising. Females are less likely to resist mating with any male who was successful in displacing another one (Kunz et al. 2021b), and in all studied populations, flanged males can consistently displace unflanged males (Utami Atmoko et al. 2009b). Thus, even though unflanged males spend more time in association with females and have higher copulation rates than the flanged males (Kunz et al. 2021b), they always risk being displaced. As a result, flanged males appear to be able to time their copulations better than unflanged males to the conception period.

Previous studies suggested that unflanged males have siring chances when flanged males do not associate and mate with a female (cf. Banes et al. 2015). Unflanged males may therefore be more likely to sire the offspring of primiparous mothers, in both semi-captivity (Tajima et al. 2018) and in natural populations (Utami Atmoko et al. 2009a). However, we did not find any evidence for this in Tuanan.

Even though newly flanged males increased involvement in physical fights (as demonstrated by a sudden increase in their injuries), we found no evidence that they have an immediate reproductive advantage over older flanged males. Based on a flanging age of approximately 25 years, we estimate that most males begin siring infants when they are at least 30 years old. This pattern deviates from that found for many other primates in which newly matured males are most likely to be dominant and sire offspring (e.g., baboons, *Papio* spp.: Altmann and Alberts 2003; crested macaques *Macaca nigra*: Marty et al. 2015; reviews: van Noordwijk and van Schaik 2004; Teichroeb and Jack 2017).

In general, the delay in flanging on Borneo is reported to be shorter than in Sumatra (Delgado and van Schaik 2000; Utami Atmoko et al. 2009b; Dunkel et al. 2013). Tuanan data suggest an approximately 10-year period of (slow) growth to reach full adult size and secondary sexual features. In contrast, where instantaneous reproductive skew among flanged males is thought to be higher, concentrated in a clearly dominant resident flanged male, such as in (northwestern) Sumatra (Utami Atmoko et al. 2002, 2009a; Lenzi 2014), relatively more unflanged males are seen, suggesting flanging to be more delayed (van Schaik 2004; Utami Atmoko et al. 2009b). This is consistent with the model by Pradhan et al. (2012) proposing a close link between monopolization of siring chances and the timing of flanging. However, while that model can explain the difference between the populations, it cannot explain why Bornean males wait around 10 years until they flange. The results reported here suggest that if they were to flange at a smaller body size, the attacks to which newly flanged males are exposed would be far more likely to be lethal.

### Paternity and residence

Similar to the low rate of siring by unflanged males, most (26 of 35 genotyped) flanged males were not assigned offspring as well. Assigned paternities in Tuanan suggest concentration in a relatively small number of flanged males, some with multiple successes over a period of at least 10 years, and without them being consistently dominant over others. Here, we explore this pattern and its consequences.

Presence in the area is obviously necessary for paternity. However, although we found a positive correlation between a male’s recorded presence in the area and his overall siring success, it was weak. For the smaller sample of male presence during known conception windows, most (7 of 8) eventual sires were seen in the area, suggesting our presence data are of sufficient quality. Thus, many residents and irregulars did not sire offspring in the area, similar to most visitors (although at least one of them was successful). Sires for at least 4 genotyped infants of regularly followed mothers could not be identified, and we cannot exclude that these infants were sired by another irregular or unrecognized visitor. In short, regular presence itself is not a sufficient condition for siring success, even among flanged males.

A male’s long calls and his response to long calls by other males are the clearest correlates of his motivation to confront other males (Spillmann et al. 2017b). This explains why female orangutans approach male long calls, interpreted not only as reflecting an attraction to flanged males as potential mates (Mitani 1985; Fox 2002) but also as seeking protection against harassment by other males (Fox 2002; van Noordwijk and van Schaik 2009; van Schaik et al. 2013). Long-call and response rates might therefore correlate with their siring success, as suggested for NW Sumatran populations, where a single flanged male may be dominant for many years (Setia and van Schaik 2007; Utami Atmoko et al. 2009b). However, in Tuanan, each male’s confidence as measured by his long-call behavior seemed to be in constant flux and largely dependent on who else was present in the area (Spillman et al. 2017b), suggesting a low long-term monopolization potential for any flanged male. In addition, our limited dataset of long-call behavior during known conception windows suggested sires were not calling or responding more than other males present and thus did not show a higher willingness to confront competitors during these periods. Thus, like presence, dominance, even if temporary, may contribute but does not strongly predict siring success in Tuanan.

Of the 4 males with multiple paternities (Fig. S4), we only have enough focal data for Niko and Otto (Fig. 5). They concentrated not only their known fertilizations but also their ranging activity mostly in a limited part of the study area, at least during their longer stays within it (when we could collect focal data on them). Thus, these males focused their reproductive efforts on a small area in which they could regularly encounter the resident females and monitor their reproductive states. Moreover, they did so consistently over many years. This resulted in at least one (and probably another) pair of full siblings and at least one mother-daughter pair with offspring sired by the same male. This pattern of fertilizations by a male concentrated in space but spread over a long time may result in clusters of female relatedness above what is expected by female philopatry alone.

Importantly, while these particular flanged males seem to concentrate their reproductive attention in limited areas, their ranges still widely overlap with each other and with those of numerous other males, and no signs of a permanent dominance hierarchy, let alone signs of territory “ownership,” could be recognized. In addition, despite semi-localized siring over extended periods, most (7/9) of the consecutive siblings with known sires have different sires, despite the regular presence in most cases of the older sibling’s sire. Finally, due to the low incidence of associations between females and flanged males (Kunz et al. 2021a), there is no behavioral evidence of consistent “friendships” between females and these sires. Thus, even though continued regular presence might allow sires to guard their mates and their infants, there is no evidence. Moreover, in most cases, some temporarily dominant males also roamed in the conceiving female’s home range. Similar local concentration was suggested to explain the spatial distribution of paternities within some chimpanzee communities (Langergraber et al. 2013), where (some) males tend to focus their associations, grooming, and mating with females ranging within a small part of their large communally defended ranges.

What made certain males more successful than many others with high or irregular presence remains unclear. Long-term resident orangutans do not divide up the space among themselves, whereas numerous other males are using the same area. However, the high degree of ranging overlap of sires and the mothers of their offspring, both before and after they give birth, may allow the long-term resident males to track the local females’ fertility status over a long time. Yet, with their extremely low close encounter rate with females, it is not clear how males update their information. From the female’s perspective, a more active role enabled by concealed ovulation (Knott et al. 2010; Durgavich 2022) would allow females to express their preference through selective proceptivity (Fox 2002; Kunz et al. 2021b) and possibly post-copulatory selection. Female sexuality in primates with a lactation/gestation ratio > 1 (van Schaik 2000) can generally be interpreted in light of the avoidance of infanticide (Hrdy and Whitten 1987; van Schaik et al. 2004; Palombit 2015). So far, infanticide is suspected but has not been observed in wild orangutans (Knott et al. 2019; Scott et al. 2019; but see Beaudrot et al. 2009), yet the extremely long period of infant dependence (van Noordwijk et al. 2018) implies vulnerability over multiple years (van Schaik 2000). Kunz et al. (2022) show how orangutan sexuality can most parsimoniously be interpreted in light of infanticide avoidance. For instance, the preference for mating with flanged males, without it being exclusive, functions to bias paternity toward males who would be more likely to benefit from infanticide if they had not mated with the female. Likewise, females are more likely to resist mating attempts when higher-ranking males are within earshot, suggesting a role in the manipulation of paternity assessments (Kunz et al. 2021b).

Females are known to resist mating attempts relatively more often when the male is a visitor or irregular (Kunz et al. 2023). Such short-term visitors, even if they are temporarily dominant around the time of conception, will only rarely be in the area once the infant is born. Even if they would occasionally return, they might not benefit from infanticide (Janson and van Schaik 2000; van Schaik 2004) because they are less likely to be present at the right moment, given that it takes on average 6 months (van Noordwijk et al. 2018) before the female is ready to conceive again. In contrast, by mating with a long-term resident flanged male, whose ranging overlaps with her whole home range, a female may benefit. First, this may increase the probability he will act as a (future) protector against other males (cf. Marzec et al. 2016). Second, and most importantly, if a female would not mate with such a male, he could pose a serious risk to commit infanticide, since he is a resident, and thus likely around by the time she would be ready to conceive again. The distribution of paternities suggests that females mate with the long-term residents in their home range and also with many others who could be occasionally present. In fact, the low rate of success of newly flanged males suggests that these may need some time to establish themselves to be perceived by females as long-term residents and then gain higher chances of siring offspring.

The concentration of paternities in the males known to be fully flanged for many years suggests increasing reproductive success with increasing age, a common pattern among large mammals with slow life history (e.g., sperm whales: Whitehead and Weilgart 2000; elephants: Hollister-Smith et al. 2007; giraffes: Castles et al. 2019; kangaroos (*Macropus giganteus*): Montana et al. 2020; and maybe dolphins (*Tursiops* sp*.*): Foroughirad et al. 2022). In addition, our results suggest that orangutan sires are on average older (over 30 years) than males of other great ape species and also have a less pronounced siring peak (Ishizuka et al. 2018; Langergraber et al. 2012; Robbins and Robbins 2018; Surbeck et al. 2017; Muller et al. 2020; Masi et al. 2021).

Finally, it remains unclear whether all males follow the same basic career trajectory of settling in a given home range after natal dispersal to become a long-term resident with an extended period of a modest level of siring success, albeit with apparently large between-male variation in actual success. The males with irregular presence and especially the visitors, some of whom show high willingness to confront other males (Spillmann et al. 2017b), point to the possibility that there is an alternative reproductive tactic. However, we cannot exclude that these irregulars and visitors of Tuanan might be successful long-term residents somewhere else, given their large ranges. After all, the Tuanan “long-term residents” are frequently absent for longer periods as well. Solving this puzzle would require sampling known males over much larger areas than currently feasible.

### Comparing populations and species

In contrast to the earlier studies in Suaq and Ketambe (both in Northwest Sumatra), where a locally dominant male appeared to sire most offspring during his tenure, we found male dominance to be in constant flux and paternities to be spread over multiple males during overlapping periods. A key difference between the known Bornean and Northwest Sumatran populations is the higher cost of association (more travel and less feeding time) for males and females, as shown by Kunz et al. (2021a) comparing Tuanan with Suaq. This higher cost apparently limits effective long-term mate guarding and thus the monopolization potential by a single (flanged) male. This difference between Tuanan and Suaq is correlated with the much lower fruit productivity in Bornean peat swamps than in the richer forests of NW Sumatran (Wich et al. 2011), which may also lead to greater fluctuations in body condition and thus in dyadic dominance. When steady food provisioning permitted a Bornean orangutan male in the Tanjung Puting release site to monopolize access to females over many years, this single male was found to sire 75% of all offspring in a study area similar in size to Tuanan (Banes et al. 2015). Thus, social organization and behavior in both Sumatran and Bornean orangutans are flexibly adjusted to local circumstances (cf. Roth et al. 2020). This suggests that differences between sites, or populations, in male mating tactics reflect plastic responses to local socio-ecological conditions (Delgado and van Schaik 2000), perhaps in interaction with genetic predispositions, given evidence for different genetic physiological adaptations between the *Pongo* species (Mattle-Greminger et al. 2018).

Finally, the pattern in male reproduction found in orangutans differs from that of the African great apes. First of all, female philopatry among the African great apes is extremely rare (Langergraber et al. 2009; Watts 2012; Sakamaki et al. 2015; Robbins et al. 2019; Masi et al. 2021). Second, although orangutans share extreme sexual dimorphism with gorillas, the orangutan mating system does not consist of sires monopolizing a local cluster of females as in gorillas (Robbins et al. 2019; Manguette et al. 2020). Third, flanged orangutan males did not show the slightest tendency to move around together in a shared range, as in male chimpanzees and bonobos (e.g., Samuni et al. 2022), although the latter differ in other aspects of their social system. Thus, each extant great ape species shows a largely or partly unique social system. Moreover, each species also shows intraspecific variation related to their ecological conditions (e.g., Watts 2012; Langergraber et al. 2013; Robbins and Robbins 2018; Morrison et al. 2022). This variability corresponds to the massive intraspecific variation in social and mating systems found in humans (Nolan and Lenski 2009; van Schaik 2016).

## Conclusion

The distribution of paternities we found can be understood as the product of a successful female tactic to cope with the high unpredictability in male dominance relationships and in male presence in their home ranges, which may cause a high risk of infanticide. The interaction between female and male interests is likely to result in higher paternity success for those males that can maintain both a long-term and consistent presence and confidence within a given female’s home range. When and where the presence and dominance of males are less stable and predictable, or food availability prevents effective male protection (cf. Fox 2002), females copulate with many males to at least reduce the risk of infanticide (Knott et al. 2010; Kunz et al. 2021b). This dispersed life style of orangutans may be unusual among primates, who tend to live in social groups with rather stable membership. However, the “solitary” females of many other mammal species are similarly confronted with a huge variation in the set of male competitors for each conception. Indeed, uneven paternity distributions are common among them too (e.g., Bellemain et al. 2006; Costello et al. 2009; Allen et al. 2015), potentially reflecting a similar compromise of male and female mating tactics.

Finally, our new understanding of orangutan male reproductive careers, albeit preliminary, should strengthen the motivation to focus conservation efforts on the protection of large areas of connected habitat to enable their natural pattern of dispersal and subsequent ranging over extended areas. This will not only benefit orangutans but other species in their ecosystem as well.

### Supplementary information

Below is the link to the electronic supplementary material.

Supplementary file1 (DOCX 4843 kb)

Supplementary file2 (DOCX 22 kb)

Supplementary file3 (XLSX 47 kb)

Supplementary file4 (DOCX 14 kb)

## Data Availability

Data is provided in the MS and supplementary material. Spatial data can be accessed on Movebank.org: https://www.movebank.org/cms/webapp?gwt_fragment=page=studies,path=study2765563865
