# Supplementary material for: Reproductive success of Bornean orangutan males: scattered in time but clustered in space
Source: Behav Ecol Sociobiol. 2023 Dec 6;77(12):134. doi: 10.1007/s00265-023-03407-6 (PMC10700224; doi:10.1007/s00265-023-03407-6)
Supplement: Supplementary file 1 — Supplementary file1 (DOCX 4843 kb) [file 265_2023_3407_MOESM1_ESM.docx]

**Electronic Supplementary material 1**

Supplementary Figures and Tables

**Reproductive success of Bornean orangutan males: spread out in time but clustered in space**

Maria A. van Noordwijk*, Laura R. LaBarge, Julia A. Kunz, Anna M. Marzec, Brigitte Spillmann, Corinne Ackermann, Puji Rianti, Erin R. Vogel, S. Suci Utami Atmoko, Michael Kruetzen, Carel P. van Schaik Behavioral Ecology and Sociobiology

*Corresponding author: vnoord@ab.mpg.de

Figure S1

Total observed presence per 3 month periods (max N=60) of all recognized flanged males in the study area in relation to the number of assigned offspring sired during the study period. The size of the bubble indicates number of identical values


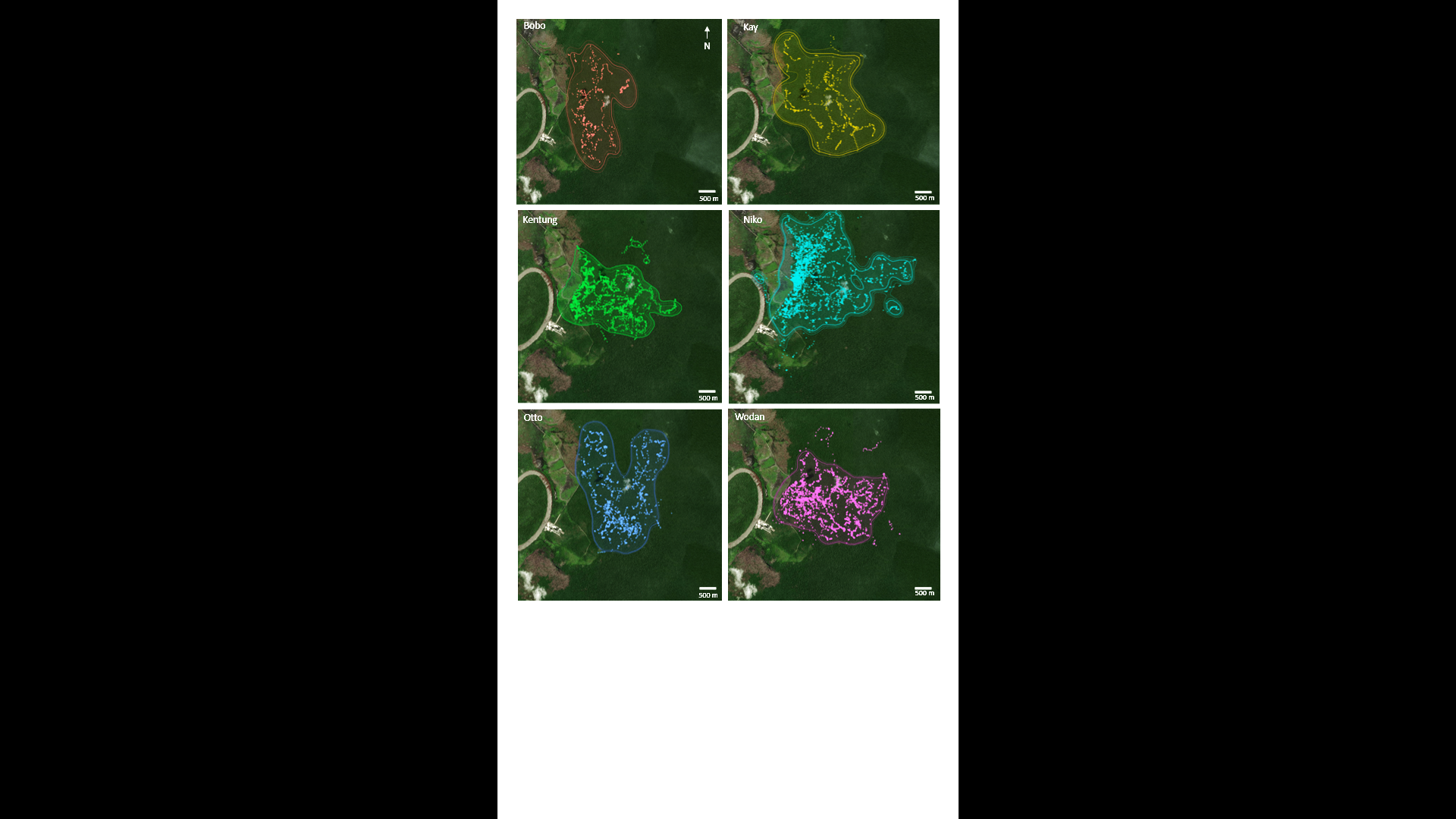


Figure S2

95% Autocorrelated kernel density Estimates (AKDE) for long-term space use by 6 sires


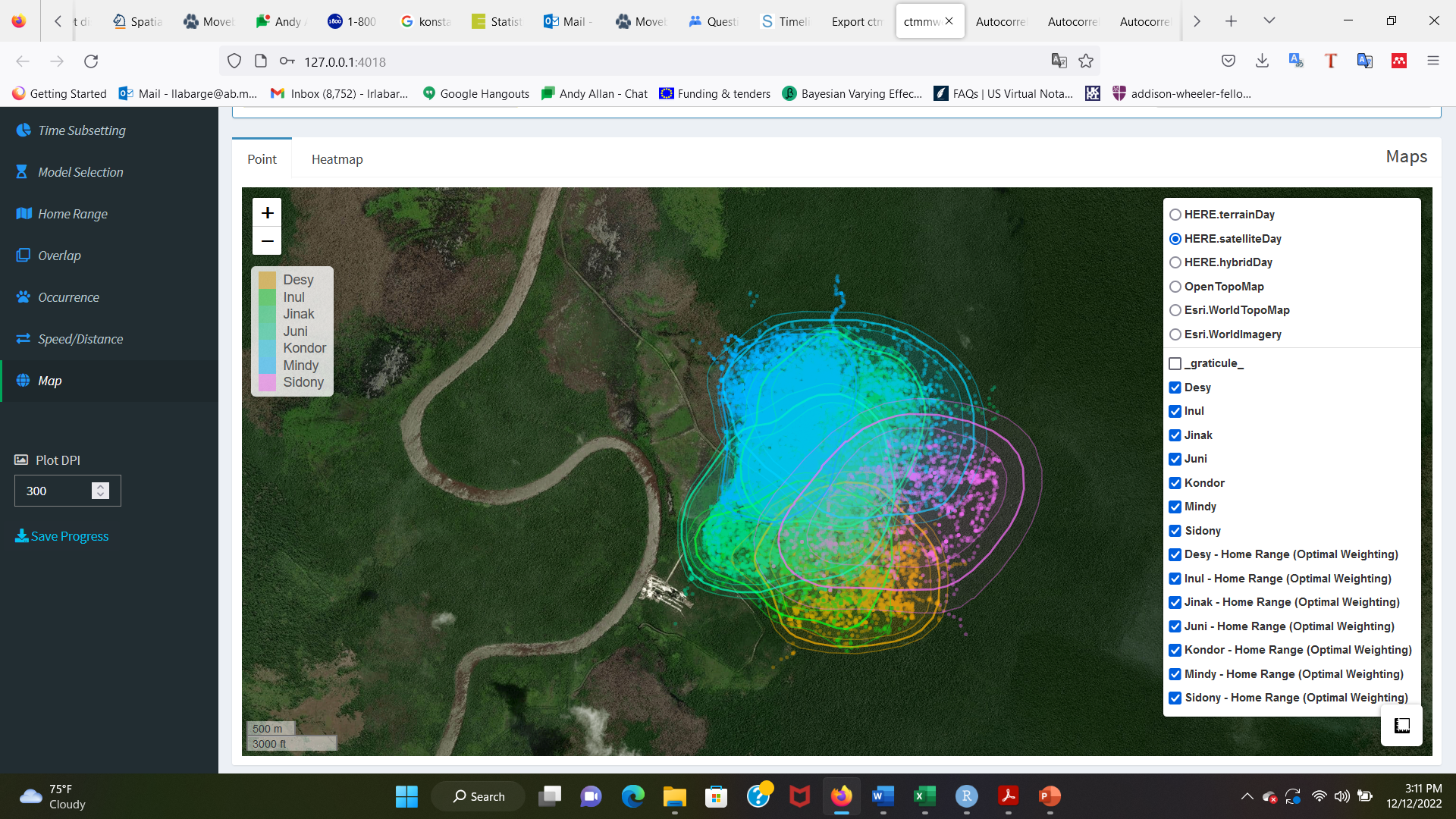


Figure S3

Overlapping ranges of the adult females (95% Autocorrelated kernel density Estimates (AKDE) for long-term space use), included in the analyses for female-sire analyses

Figure S4

Map of the Tuanan study area with, for each male, the location of the core of the natal ranges of his assigned offspring’s mother: e.g. N1 is first offspring of Niko (see figure 1) etc. (Note for Guapo 1 and Kentung 1 mother is unknown, most likely ranging outside the study area.). G4, born in 2008, paternity based on only 12 matched loci (but no mismatches), not included in high confidence paternities in the text

Figure S5

Overlap in AKDEs for sire-mother dyads for 3 year periods before and following a birth measured via the Bhattacharyya Coefficient estimates (see Table S7)


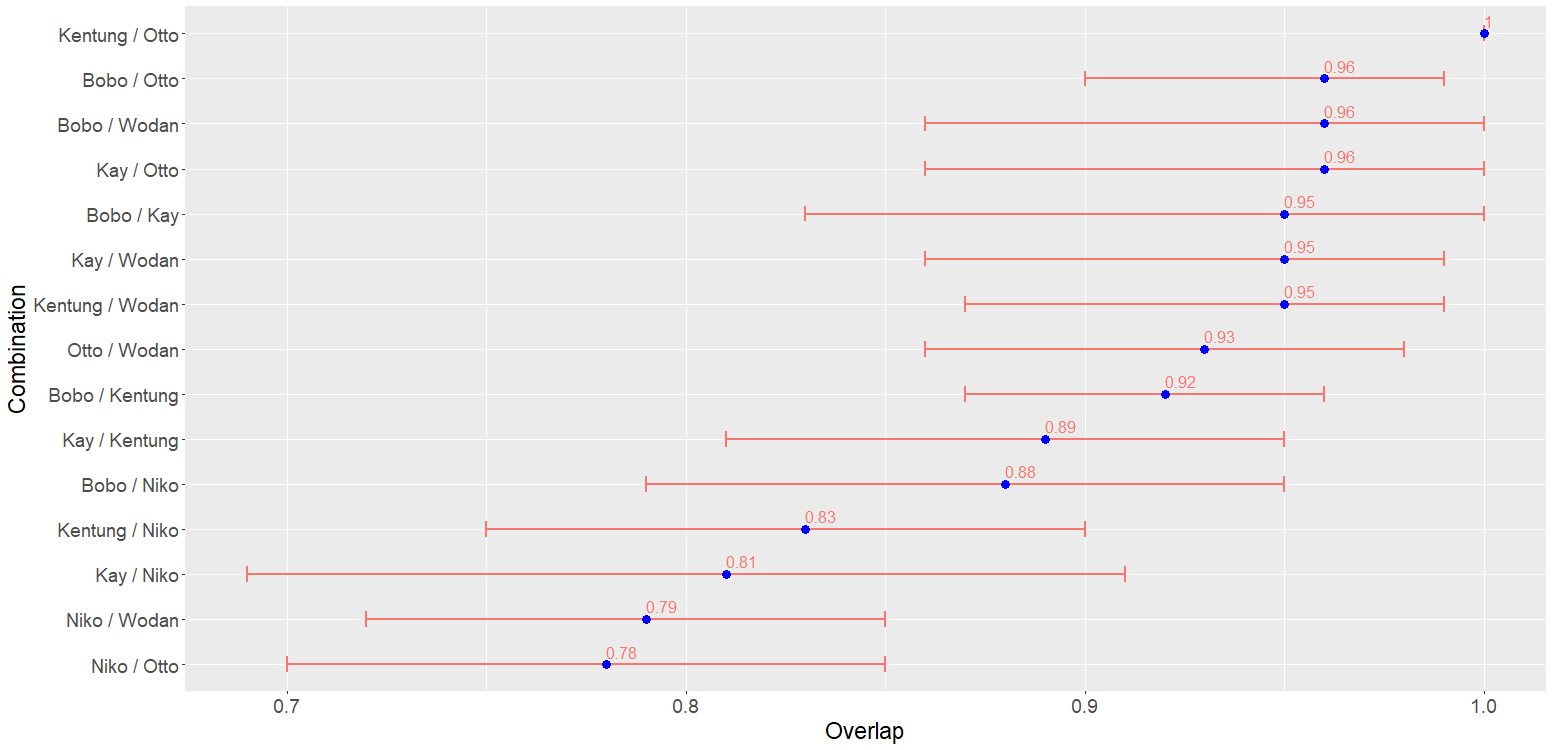


Figure S6

Long-term overlap of space use of known sires calculated via the Bhattacharyya Coefficient (BC): values are based on variation in intensity of use across the AKDE utilization distribution. These values do not account for the fact that males will use similar areas at different time, thus they do not reflect more fine-scale spatiotemporal avoidance strategies

Table S1

The number of focal follows (July2010-July2018) of identified adult females and males (flanged and unflanged) that were maintained until the focal made a night nest or were ‘lost’ because the focal left the study area, fled after encountering another orangutan, or moved fast on the ground without obvious social trigger, or ‘other’ reason (including weather, bee attack, observer decision).

| SUMMARY TABLE Follows LOST or to NEST | | | |  |  | |  |  |
| --- | --- | --- | --- | --- | --- | --- | --- | --- |
|  | to NEST | LOST | **TOTAL** | Out of area | | ground | social | other |
| adult female | 1802 | 93 | **1895** | 26 | | 11 | 9 | 47 |
| flanged male | 1377 | 263 | **1640** | 57 | | 66 | 69 | 71 |
| unflanged male | 318 | 55 | **373** | 11 | | 10 | 22 | 12 |
| TOTAL | 3497 | 411 | **3908** | 94 | | 87 | 100 | 130 |

Difference between adult classes in getting lost during focal follow

|  |  | Chi SQ | P | |  |
| --- | --- | --- | --- | --- | --- |
| lost: total |  |  |  | |  |
| adf-flm |  | 119 | <0.0001 | *** | |
| adf-ufm |  | 47.85 | <0.0001 | *** | |
| flm-ufm |  | 0.29 | 0.59 | NS | |
|  |  |  |  |  | |
| lost: out of area |  |  |  |  | |
| adf-flm |  | 16.06 | <0.0001 | *** | |
| adf-ufm |  | 3.9 | 0.048 | * | |
| flm-ufm |  | 0.12 | 0.729 | NS | |
| females-males |  | 15.89 | <0.0001 | *** | |
|  |  |  |  |  | |
| lost: on ground |  |  |  |  | |
| adf-flm |  | 47.33 | <0.0001 | *** | |
| adf-ufm |  | 12.79 | 0.0003 | *** | |
| flm-ufm |  | 1.16 | 0.28 | NS | |
|  |  |  |  |  | |
| lost: social lost |  |  |  |  | |
| adf-flm |  | 55.04 | <0.0001 | *** | |
| adf-ufm |  | 64.03 | <0.0001 | *** | |
| flm-ufm |  | 1.64 | 0.2 | NS | |

Table S2

Comparison of the frequency of ‘encounters’ with females by flanged and unflanged males: the number of male focal follow days on which the male was within 50m of a female for any time (‘in association’). Only included are follows lasting at least 3 h for males who were followed on at least 10 different days

| Male morph | Total N days with female encounter | # days without female encounter | # follow days | % days with female encounter |
| --- | --- | --- | --- | --- |
| Flanged (N=31) | 380 | 1609 | 1989 | 19.1 |
| Unflanged (N=18) | 383 | 371 | 754 | 50.8 |

Table S3

Male presence per individual and his morph at the time during the 6-months conception windows for 8 genotyped infants with assigned sires indicated in shading (Fla for flanged males and unfl for unflanged males). Conception windows are defined as 4 months before estimated conception and 2 months into pregnancy

Table S4

Long-term sampling schedule of ranging data (one point per half hour focal follow) for the 4 sires and 6 females they successfully mated with

| Name | Sex | Date - First Follow | Date - Last Follow | Duration (months) | # Location Points |
| --- | --- | --- | --- | --- | --- |
| Jinak | Female | 2003-08-01 | 2017-02-17 | 168 | 11452 |
| Mindy | Female | 2003-09-01 | 2018-05-12 | 182 | 14006 |
| Juni | Female | 2006-02-10 | 2018-06-09 | 152 | 9442 |
| Inul | Female | 2003-10-24 | 2017-05-06 | 167 | 2590 |
| Desy | Female | 2003-12-01 | 2018-05-10 | 179 | 4164 |
| Juni | Female | 2006-02-10 | 2018-06-09 | 152 | 9442 |
| Kondor | Female | 2006-03-08 | 2018-02-14 | 148 | 3094 |
| Niko | Male | 2003-09-10 | 2016-12-19 | 159 | 4510 |
| Bobo | Male | 2004-02-14 | 2016-02-24 | 144 | 884 |
| Kay | Male | 2005-03-10 | 2014-07-11 | 115 | 1025 |
| Otto | Male | 2008-08-03 | 2016-09-25 | 101 | 2086 |

Table S5

Allele frequency analysis Cervus 3.0.7

| Locus | k | N | HObs | HExp | PIC | NE-1P | NE-2P | NE-PP | NE-I | NE-SI | HW | F(Null) |
| --- | --- | --- | --- | --- | --- | --- | --- | --- | --- | --- | --- | --- |
| D1S550 | 9 | 119 | 0.681 | 0.711 | 0.660 | 0.698 | 0.527 | 0.339 | 0.133 | 0.429 | NS | 0.0190 |
| D2S1326 | 8 | 122 | 0.549 | 0.585 | 0.512 | 0.822 | 0.684 | 0.527 | 0.244 | 0.520 | NS | 0.0354 |
| D3S2459 | 9 | 120 | 0.825 | 0.821 | 0.792 | 0.541 | 0.365 | 0.189 | 0.059 | 0.356 | NS | -0.0073 |
| D4S2408 | 5 | 107 | 0.757 | 0.696 | 0.642 | 0.723 | 0.554 | 0.374 | 0.146 | 0.440 | NS | -0.0487 |
| D5S1470 | 6 | 118 | 0.475 | 0.487 | 0.439 | 0.876 | 0.734 | 0.579 | 0.311 | 0.585 | NS | 0.0080 |
| D13S321 | 6 | 125 | 0.560 | 0.647 | 0.585 | 0.775 | 0.617 | 0.446 | 0.186 | 0.474 | NS | 0.0763 |
| D13S765 | 5 | 123 | 0.642 | 0.660 | 0.604 | 0.759 | 0.593 | 0.415 | 0.170 | 0.464 | NS | 0.0121 |
| D16S420 | 7 | 122 | 0.566 | 0.544 | 0.487 | 0.845 | 0.699 | 0.539 | 0.265 | 0.545 | NS | -0.0222 |
| D2S141 | 11 | 120 | 0.800 | 0.778 | 0.740 | 0.611 | 0.433 | 0.248 | 0.085 | 0.384 | NS | -0.0153 |
| D5S1505 | 12 | 115 | 0.800 | 0.814 | 0.788 | 0.536 | 0.361 | 0.176 | 0.058 | 0.360 | NS | 0.0049 |
| D6S501 | 9 | 121 | 0.702 | 0.700 | 0.657 | 0.706 | 0.529 | 0.336 | 0.132 | 0.435 | NS | 0.0017 |
| D4S1627 | 7 | 106 | 0.689 | 0.663 | 0.613 | 0.753 | 0.582 | 0.399 | 0.163 | 0.461 | NS | -0.0253 |
| O4A5 | 6 | 109 | 0.661 | 0.621 | 0.585 | 0.776 | 0.596 | 0.398 | 0.179 | 0.486 | NS | -0.0427 |
| O4B5 | 8 | 107 | 0.720 | 0.705 | 0.655 | 0.709 | 0.536 | 0.351 | 0.136 | 0.433 | NS | -0.0137 |
| O4_6 | 3 | 117 | 0.615 | 0.611 | 0.538 | 0.815 | 0.671 | 0.521 | 0.224 | 0.502 | NS | -0.0058 |
| D5S1457 | 9 | 120 | 0.817 | 0.776 | 0.736 | 0.624 | 0.446 | 0.266 | 0.089 | 0.386 | NS | -0.0294 |
| O4A1 | 5 | 111 | 0.802 | 0.752 | 0.703 | 0.669 | 0.493 | 0.318 | 0.109 | 0.403 | NS | -0.0353 |
| O4B17 | 8 | 109 | 0.734 | 0.794 | 0.762 | 0.582 | 0.403 | 0.217 | 0.072 | 0.373 | NS | 0.0415 |
| O4C13 | 6 | 93 | 0.591 | 0.591 | 0.502 | 0.820 | 0.697 | 0.549 | 0.256 | 0.520 | NS | -0.0015 |
| O4C9 | 5 | 112 | 0.643 | 0.700 | 0.642 | 0.727 | 0.560 | 0.385 | 0.147 | 0.438 | NS | 0.0439 |

**** Summary statistics ****

Number of individuals: 131

Number of loci: 20

Mean number of alleles per locus: 7.200

Mean proportion of loci typed: 0.8763

Mean expected heterozygosity: 0.6829

Mean polymorphic information content (PIC): 0.6320

Combined non-exclusion probability (first parent): 0.00110415

Combined non-exclusion probability (second parent): 0.00000489

Combined non-exclusion probability (parent pair): 1.201E-0009

Combined non-exclusion probability (identity): 1.121E-0017

Combined non-exclusion probability (sib identity): 0.00000009

Table S6

Cervus 3.0.7 parentage analyses results, reporting only values with >95% confidence

**

Table S7

Pairwise distributional range overlap of female-male dyads, based on AKDEs (autocorrelated kernel density estimates) calculated via the Bhattacharyya Coefficient (BC), the expected overlap from female and male perspective based on the medians for all range-resident members of the opposite sex, and the percentage of each individual’s range covered by the area of overlap with the mate

| Pair | | |  | |  | |  | |  |  |  |
| --- | --- | --- | --- | --- | --- | --- | --- | --- | --- | --- | --- |
| Female | Male | Bhattacharyya Coefficient - Distribution Overlap (95% CIs) | | Expected Overlap (female perspective) | | Expected Overlap (male perspective) | | % Range covered by area of overlap (female) | | % Range covered by area of overlap (male) | |
| Inul | Bobo | 0.76 (0.69 - 0.81) | | 0.68 | | 0.58 | | 100 | | 22 | |
| Desy | Otto | 0.73 (0.59 -0.85) | | 0.55 | | 0.49 | | 100 | | 27 | |
| Jinak | Otto | 0.61 (0.51 - 0.71) | | 0.70 | | 0.49 | | 100 | | 32 | |
| Kondor | Otto | 0.64 (0.52 - 0.78) | | 0.81 | | 0.49 | | 92 | | 53 | |
| Juni | Kay | 0.70 (0.61 - 0.78) | | 0.79 | | 0.49 | | 93 | | 33 | |
| Mindy | Niko | 0.83 (0.78 - 0.87) | | 0.59 | | 0.39 | | 100 | | 26 | |
| Jinak | Niko | 0.64 (0.58 - 0.70) | | 0.70 | | 0.39 | | 91 | | 32 | |

Table S8

Overlap in AKDEs for sire-mother dyads for 3 year periods before and following a birth measured via the Bhattacharyya Coefficient estimates

|  |  | Pre-Birth |  | Post-Birth | |
| --- | --- | --- | --- | --- | --- |
| Infant | Dyad | Est. | CI. | Est. | CI. |
| Mawas | Mindy + Niko | 0.85 | (0.74, 0.93) | 0.77 | (0.69, 0.84) |
| Joya | Jinak + Otto | 0.49 | (0.11, 0.94) | 0.75 | (0.50, 0.94) |
| Kilmino | Kondor + Otto | 0.79 | (0.61, 0.93) | 0.51 | (0.36, 0.67) |
| Ivan | Inul + Bobo | 0.90 | (0.69, 0.99) | 0.71 | (0.38, 0.96) |
