# Supplementary material for: Reproductive success of Bornean orangutan males: scattered in time but clustered in space
Source: Behav Ecol Sociobiol. 2023 Dec 6;77(12):134. doi: 10.1007/s00265-023-03407-6 (PMC10700224; doi:10.1007/s00265-023-03407-6)
Supplement: Supplementary file 2 — Supplementary file2 (DOCX 22 kb) [file 265_2023_3407_MOESM2_ESM.docx]

**Electronic Supplementary Material 2**

Supplementary text: genetics

**Reproductive success of Bornean orangutan males: spread out in time but clustered in space**

Maria A. van Noordwijk*, Laura R. LaBarge, Julia A. Kunz, Anna M. Marzec, Brigitte Spillmann, Corinne Ackermann, Puji Rianti, Erin R. Vogel, S. Suci Utami Atmoko, Michael Kruetzen, Carel P. van Schaik Behavioral Ecology and Sociobiology

*Corresponding author: vnoord@ab.mpg.de

***Haplotypes***

Fourteen different mtDNA haplotypes were found in the Tua

nan population, including four not yet reported by Arora et al. (2012) for the same population. Most females with offspring had the same common haplotype as most of the males; however, three haplotypes were almost exclusively found in adult females and their offspring, with only a single unflanged male with the same rare haplotype as two females seen at the northern edge of Tuanan. In addition, two nulliparous females had a distinctly different haplotype, only one of these was shared with seven adult males. Among adult males we found eight additional haplotypes, six of these were only found in a single individual. This sex difference in distribution of mtDNA haplotypes is consistent with male dispersal and female philopatry, but with some female exploration or forced dispersal (cf. Arora et al. 2012; Nietlisbach et al. 2012; Ashbury et al. 2020).

**Parentage analyses**

In the *maternity analysis* (using a minimum of 15 shared loci for the dyad), known mothers were correctly assigned in 20 cases (whereas 3 with fewer loci were also assigned as expected). For 5 immatures an adult female relative (sister, grandmother, based on demographic data) was assigned by the program (Cervus 3.0.7) as the most likely mother, despite at most 1 mismatch with the known mother from field observations. These were all members of the largest matriline including 6 adult female relatives in the sample (matriarch with 3 parous daughters and 2 parous granddaughters). A similar confusion was apparent in a matriline with 3 adult females ranging at Tuanan’s periphery. In another two cases a mix-up in the field could not be excluded and dependents were assigned to another not well-known mother ranging in the same area. For subsequent paternity analyses we included the known mother from field observations in the mother-offspring-sire trio in all cases.

For adult females not previously known as dependents during the study, we identified eight mother-daughter dyads, four of which had previously been genetically identified by Arora et al. (2012). For the four new adult-adult dyads, limited field observations support their assignment, in that they seem to have partially overlapping ranges, and in one dyad we also observed non-agonistic associations, similar to those seen among other female relatives (cf. van Noordwijk et al. 2012, Ashbury et al. 2020), but some females were seen in the area too rarely for a corroborating social assessment.

For 1 adolescent and 6 additional nulliparous females the genetic mother was not found among the females sampled in Tuanan. Three of these ‘new’ females, were seen only in one month, the others with irregular intervals. These young females may have been in the exploratory phase of their range establishment, assuming their mothers live(d) in an adjacent area (cf. Ashbury et al. 2020), or they may have been displaced from their natal area by forest destruction. Four of these young females plus the adolescent have the common haplotype A, suggesting a nearby natal area, but two have a distinct maternal haplotype, potentially suggesting a more distant origin.

At least 65% (17 of 26) of the genotyped parous females were assigned as the daughter or mother of at least one other known adult female at least partially ranging within Tuanan, consistent with female philopatry. In contrast, only one young unflanged male was assigned as the son of a known female, with a peripheral home range. This male was only seen for a short period and probably subsequently dispersed. None of the other adult males, flanged or unflanged, was the apparent son of one of the known parous females, consistent with male dispersal during sub-adulthood.

To assess the likelihood that first degree relatives in the sample would affect *paternity assignments,* as we found for maternity analyses, we checked for the presence of father-son dyads among the adult males seen in the area. This paternity analysis of all possible male-male dyads (N=62 males; excl. males with a known local mother), yielded only 2 sire-son dyads at 95% confidence: one older flanged male with a young unflanged male (son of a mother with a peripheral home range), who was only seen in the area during a few years and may have dispersed since; the other dyad consists of two flanged males who were both rarely seen and neither was assigned as possible sire to any known offspring (see details Table ESM2). Based on this, we ignore the presence of possible close male relatives as a source of error in our paternity analyses of immatures with known mothers.

*Table ESM2*

Potential sire-son dyads (>80% confidence)

| male 1 | morph male1 | male 2 | morph male 2 | Confidence % | Y-match | Father-son? |
| --- | --- | --- | --- | --- | --- | --- |
| **Kentung** | **Flanged <2004** | **Nanio** | **small unflanged** | **95** | **yes** | **likely** |
| **Sultan** | **Flanged <2005** | **MLG** | **unflanged** | **95** | **unk** | **maybe** |
| Wodan | flanged 2010 | Kiki | unflanged | 80 | no | no |
| Henk | flanged <2004 | Vladimir | flanged 2010-2017 | 80 | yes | maybe |
| Vini | unflanged | Pilatus | unflanged | 80 | yes | Unlikely* |
| Henk | flanged <2004 | Rex | flanged <2008 | 80 | unk | maybe |
| Anton | flanged 2018 | Pri | unflanged | 80 | unk | Unlikely* |
| Fugit | flanged <2004 | Zeke | flanged <2004 | 80 | unk | maybe |
| Tarzan | flanged <2013 | Utli | small unflanged | 80 | unk | maybe |

*Unlikely sire-son dyads since these males are of very similar size/estimated age (both known as unflanged)

**Lower confidence paternity assignments**

Some parent-offspring relationships were assigned with lower confidence (80-95%) than the threshold used in the main text. This lower confidence was due to fewer than 15 matched loci for the parent-offspring pair, more than 2 mismatches or many shared common alleles. If we include these lower confidence relationships, we add one mother-adult daughter pair, resulting in 73% (19 out of 26) of the adult females having at least one adult maternal relative in the area. We also would add 5 additional sire-offspring-mother trios: 2 by unflanged males, 1 by a male with unknown morph at the time of conception and 1 most likely flanged (after siring three other offspring before 2003. This last one suggests there is a second full sibling pair (mixed sex) in the study population.

The potential paternities by unflanged males were both with parous females who had raised offspring successfully before. In one case the male had been known for many years and was fully flanged 3 years later, the other male was frequently seen throughout the study and flanged 2 years later. However, this conception happened during a period with multiple ‘new’ flanged males in the area, which could not all be sampled sufficiently to be included in the analyses.

Including the 3 conception windows with a low confidence sire assignment (with known morph) would result in on average 10.7 ± 3.5 flanged and 6.4 ± 3.1 unflanged males for a total of 17.1 ± 6.3 identified males per conception in the area. Per flanged male present per conception window this results in 0.058 sirings, more than twice the rate for unflanged males (0.024), still a non-significant difference (Kolmogorov-Smirnov = 0.172, N=10, NS).

In conclusion, inclusion of these low confidence paternities would not affect our main conclusions: unflanged males can and do sire some offspring but at a lower rate relative to their presence during conception windows, than flanged males do. Due to some spatial concentration of paternities, full siblings may not be as uncommon as expected based on the 7.5 year birth intervals and large number of different mates per conception.
