# Supplementary material for: Reproductive success of Bornean orangutan males: scattered in time but clustered in space
Source: Behav Ecol Sociobiol. 2023 Dec 6;77(12):134. doi: 10.1007/s00265-023-03407-6 (PMC10700224; doi:10.1007/s00265-023-03407-6)
Supplement: Supplementary file 4 — Supplementary file4 (DOCX 14 kb) [file 265_2023_3407_MOESM4_ESM.docx]

**Electronic Supplementary material 4**

R-Script

**Reproductive success of Bornean orangutan males: spread out in time but clustered in space**

Maria A. van Noordwijk*, Laura R. LaBarge**, Julia A. Kunz, Anna M. Marzec, Brigitte Spillmann, Corinne Ackermann, Puji Rianti, Erin R. Vogel, S. Suci Utami Atmoko, Michael Kruetzen, Carel P. van Schaik

Behavioral Ecology and Sociobiology

*Corresponding author: [vnoord@ab.mpg.de](mailto:vnoord@ab.mpg.de)

** For details on this script please contact Dr.Laura LaBarge: lrlabarge@gmail.com

R-Script for reproducing home ranges:

library(move)

library(readr)

library(ctmm)

# log into movebank for file

# movebank contains all the GPS data and a separate file

# named "Bornean Orangutan 2012" was batched edited to contain only that year

login <- movebankLogin(username="XXXX", password="XXXXX")

pairs <- getMovebankData(study=" Reproductive success of Bornean orangutan males: spread out in time but clustered in space", login=login)

# create as.telemetry object for ctmm models

orangutan_pairs<-as.telemetry(pairs, projection = "+init=epsg:32750 +proj=utm +zone=50 +units=m +south")

#run continuous-time movement models, select the best for each individual

# then use the best model to run autocorrelated KDEs

SVF <- list()

for(i in 1:length(orangutan_pairs)){

print(i)

SVF[[i]] <- variogram(orangutan_pairs[[i]])}

names(SVF) <- names(orangutan_pairs)

## fit models with a 30 meter error to account for GPS / human error in rainforest habitat

pair.FIT <- list()

for(i in 1:length(orangutan_pairs)){

print(i)

GUESS <- ctmm.guess(orangutan_pairs[[i]],CTMM=ctmm(error=30), interactive=FALSE)

pair.FIT[[i]] <- ctmm.select(orangutan_pairs[[i]],GUESS, verbose=TRUE,trace=2)

}

names(pair.FIT) <- names(orangutan_pairs)

save(pair.FIT, file="pairFIT.Rdata")

# extent based on Tuanan map

library(raster)

Tuanan_r<-raster()

extent(Tuanan_r)<-extent(212812, 219348, 9758957, 9773670)

res(Tuanan_r)<-25

projection(Tuanan_r) <- "+init=epsg:32750 +proj=utm +zone=50 +units=m +south"

AKDE <- list()

for(i in 1:length(orangutan_pairs)){

print(i)

AKDE[[i]] <- akde(orangutan_pairs[[i]],pair.FIT[[i]][[1]], grid=Tuanan_r, weights=TRUE)

}

names(AKDE) <- names(orangutan_pairs)
